# Perceptions and digitalisation of outbreak management in UK health services: A cross-sectional survey

**DOI:** 10.1177/17571774241239221

**Published:** 2024-03-19

**Authors:** Matthew Wynn

**Affiliations:** School of Health and Society, 7046 University of Salford, Salford, UK

**Keywords:** Outbreak management, processes, perceptions, preparedness, resilience

## Abstract

**Background:**

Global challenges arise from infectious diseases which represent significant challenges to the provision of healthcare, requiring efficient management procedures to limit transmission. Evaluating current outbreak management processes within UK healthcare services is essential for identifying strengths, weaknesses, and potential improvements.

**Objectives:**

This study aimed to assess infection prevention and control (IPC) practitioners' access to outbreak management (OM) data. Secondary objectives involved determining IPC practitioners’ perceptions of outbreak management processes and the state of digitalisation of OM in the UK.

**Methods:**

National cross-sectional survey data were collected to evaluate current outbreak management approaches. To supplement this, information requests were sent to the 10 largest teaching and research NHS hospital trusts in England.

**Findings:**

The survey received 55 responses with 53 considered for analysis. Out of 10 NHS trusts, nine provided completed FOI responses, while one was unable to provide data.

**Discussion:**

The study offers unique insights into prevailing outbreak management practices within UK health services. Although positive perceptions surround key outbreak management stages, concerns arise, including varying confidence levels in surveillance processes' robustness, efficacy of management interventions, and communication effectiveness.

**Conclusions:**

The study highlights challenges with OM processes in the UK, including issues like poor surveillance and delayed outbreak detection. Positive practitioner perceptions contrast with concerns over data collection, follow-up, and limited digitalisation, relying on basic tools like Excel and Word, hindering retrospective learning.

## Introduction

### Background

Outbreaks of infectious diseases pose significant challenges to healthcare systems worldwide, requiring effective and timely response strategies to mitigate impact on public health. Infection prevention and control (IPC) and public health practitioners in the UK play a crucial role in managing outbreaks and safeguarding the population from the spread of communicable diseases. To ensure the effectiveness of existing outbreak management (OM) processes, it is necessary to assess the current state of processes within the UK healthcare system. IPC practitioners play a pivotal role, working to prevent the transmission of infections and maintain nationally mandated standards of patient safety (Health and Social Care Act, 2012). Limited research has been conducted to comprehensively evaluate the availability of key outbreak management data and the efficacy of current approaches within the UK. The author is not aware of any studies or reviews of the processes used to manage outbreaks of communicable diseases in the UK. Existing evidence points towards inconsistencies within this area of practice (Centre for Workforce Intelligence, 2015; Hale et al., 2015). Notably, a cross-government exercise to test the UK’s response to a serious influenza pandemic that took place in October 2016 involving more than 950 people concluded that:

‘the UK’s preparedness and response, in terms of its plans, policies and capability, is currently not sufficient to cope with the extreme demands of a severe pandemic that will have a nation-wide impact across all sectors’ (Exercise Cynus Report, 2017, page 6)

The lack of regulation and information about the current size, nature, and practices of the infection control workforce in the UK (CWI, 2015) may explain the paucity of data indicating how highly specialised processes such as outbreak management are conducted and could therefore be improved upon. Understanding the access to and understanding of key outbreak management processes among IPC practitioners is essential for identifying potential barriers to effective outbreak response. Resource availability, including outbreak protocols, surveillance requirements, and outbreak investigation reports, is vital for informed decision-making, prompt action, and coordination of resources. Assessing practitioners' perceptions of the efficacy of current local approaches to OM is crucial for improvement and optimisation of outbreak response strategies. Experienced IPC practitioners possess valuable knowledge and insight into the strengths and weaknesses of existing practices. Their perspectives can help identify areas of improvement, guide policy decisions, and inform future strategies to enhance outbreak management processes.

## Purpose

The research question for this study was:

• How do UK IPC services currently manage outbreaks of communicable disease?

Objectives:

(1) Determine the perceptions of UK-based IPC practitioners on the efficacy of current approaches to OM in their respective organisations.
(2) Determine the accessibility of key data to support OM efforts.
(3) Determine the current state of digitalisation of OM processes in UK health services.

By conducting a widely distributed cross-sectional survey of UK-based IPC practitioners and incorporating data obtained from Freedom of Information (FOI) requests, this study aimed to fill the existing knowledge gap by exploring both the access to key outbreak management data and the perceptions of IPC practitioners regarding the effectiveness of current approaches. This comprehensive approach provides an insight into the current state of outbreak management in the UK health services.

## Methods

### Study design

The study utilised two data collection methods. These included a cross-sectional survey and freedom of information requests to leading NHS trusts. The survey tool utilised 27 questions including demographic information and general questions exploring the availability of key surveillance functions within the respondent’s services. This was followed by Likert scale questions exploring perceptions of each aspect of outbreak management processes. The outbreak management process comprises key stages (Sistrom and Hale 2006, Torok et al., 2016) which were used as the focus of attitude scales. Firstly, the ‘Identification’ stage, involving identification of infection rates deviating from what is expected, identified through surveillance or clinical observations. Secondly, the ‘Surveillance’ stage includes collecting samples and interviewing potentially affected patients to understand the nature of the outbreak. In the ‘Case Definition’ stage, healthcare professionals collaborate to create a precise description of affected patients based on specific criteria. ‘Control Measures’ involve implementing interventions to halt the outbreak’s transmission. Lastly, effective ‘Communication’ describes the dissemination of outbreak information to stakeholders, patients, and staff, ensuring swift and accurate updates. A free-text question was also included to allow respondents to provide additional details.

In addition to the survey, FOI requests were sent to the Shelford Group, the 10 largest and leading NHS trusts. The structure of this request was based on the research objectives and sought to determine what data NHS organisations held, to understand how they managed, and reviewed their performance of outbreak management. The use of FOI requests is recommended to access data which is not otherwise disclosed (Fowler et al., 2013). Guidance provided by the University College London (2012) on FOI for academic researchers informed the methodology. In cases where data was initially withheld, FOI officers were informed of the purpose of the request to encourage a response. The request was made using explicit questions including example responses where relevant, a data collection table was also provided to facilitate the request. The time limit (18 h) within which FOI requests must be fulfilled provided insights into not only what data was available, but also how rapidly these data could be provided. The data requested included:

• The number of communicable disease outbreaks (by organism/resistance mechanism) that have occurred within the trust over the last 2 years.
• For the most recent outbreaks:
  ○ the number of staff and patients involved.
  ○ interventions which were put in place.
  ○ how the effectiveness of each intervention was evaluated.
• The digital tools used as part of the outbreak management process.

As per the UCL (2012) guidance, this study did not rely solely on FOI data and instead this was used to triangulate cross-sectional survey data to achieve a more complete picture of outbreak management processes in the UK. Importantly, this research aimed not to single out specific NHS trusts regarding their outbreak management procedures, but rather to provide insights into current practices that can inform and support investment into research and development of outbreak management practices. Consequently, individual trusts are not identified within the analysis. Freedom of Information (FOI) requests used to collect data are considered public records, reinforcing the transparency and openness of the study.

### Data collection methods

The survey tool was piloted with responses from five IPC specialists who responded to the survey then provided feedback on the content and wording of the survey. This pilot identified only minor issues with the demographic elements and as such the pilot data were included in the final analysis as no significant changes were made to the survey tool. The demographics of the pilot sample included a majority (*n* = 3) who had over 10 years of experience in IPC with the remainder having between 5 and 10 years of experience in IPC (*n* = 2). The age of the respondents ranged from 35 to 64. The majority were female (*n* = 3) with the remainder being male (*n* = 1) or preferring not to say (*n* = 1).

### Sample characteristics

The target population for the survey included infection control practitioners of any professional background who had experience of managing outbreaks of communicable disease. There is currently no data available on the number of IPC practitioners working within the NHS according to the most recent Center for Workforce Intelligence report (2015). The report also notes the membership of the Infection Prevention Society, which was over 2000 as of 2023. However, this membership will include individuals not involved in the operational management of outbreaks including researchers, commercial representatives, and link workers with no direct responsibility for managing outbreaks.

Due to the limited sample sizes and unclear overall size of the study population, performing a power calculation to determine the statistical significance of the survey responses was not possible. The survey was therefore closed after a predetermined period of 8 months.

### Survey/FOI request

The survey was conducted digitally using the JISC online survey tool. FOI requests were sent via the relevant pathways as required by each NHS organisation.

### Administration

The survey data collection period was 8 months from January to August 2023. The survey was distributed via established UK-based professional networks for practitioners working in the field of infection control. These included the Infection Prevention Society, the Hospital Infection Society and the Queen’s Nursing Institute Infection Prevention and Control Champions Network and UK-based higher education courses related to infection control (identified via IPS UK IPC courses list published online). A QR code link to the survey was also shared by the author during talks provided during the survey period. In addition, a social media advert was shared by the research team on social media, Twitter and LinkedIn, inviting individuals meeting the inclusion criteria to contact the author to receive a survey link. The survey link was not published directly in any public forums to ensure that only responses from practising professionals were considered.

### Ethical considerations

This research was given a favourable ethical opinion by the University of Salford Ethics Committee.

The survey was conducted anonymously, and no personal identifiable data was collected within the tool.

### Statistical analysis

Data was analysed using descriptive statistics and content analysis (Vaismoradi et al., 2013). Likert scale responses were analysed using Cronbach’s alpha coefficient (Tavakol & Dennick, 2011). Statements grouped into scales representing the five aforementioned stages of outbreak management, for example, ‘identification of outbreaks’, ‘surveillance’. The Cronbach’s alpha coefficient provides insight into the consistency of responses (Eisinga et al., 2013) related to each stage of the outbreak management process which is useful for indicating which aspect(s) of the OM process IPC practitioners have negative/positive perceptions of based on their current practice. The coefficient was calculated using SPSS version 29.01.1 (171). A Cronbach’s alpha coefficient of 1 indicates high internal reliability of a scale, and a coefficient of 0 indicates no internal reliability. Due to the pragmatic realities of research in small, hard-to-reach populations of unknown size such as the UK IPC practitioner population, additional tests for reliability such as criterion or construct validity were not conducted.

As the target population size is unknown and the survey was distributed via multiple channels, it is unknown how many people received access to the link. As such calculation of a non-response rate is not feasible.

## Results

### Respondent characteristics

The full details of respondent demographics can be seen in Supplementary Information 1. Respondents were predominantly between 35 and 54 years old (*n* = 28, 75.5%), with 37.7% (*n* = 20) in both the 45–54 and 55–64 age groups. The majority (*n* = 30, 56.6%) had over 10 years of IPC experience, while 24.5% had 5–10 years and 18.9% (*n* = 10) had 1–5 years. Formal qualifications included master’s degrees (*n* = 9, 17.0%) and study at master’s level (*n* = 8, 15.1%). Participants without IPC qualifications account for 20.8% (*n* = 11). Female respondents made up 88.7% (*n* = 47), males 9.4% (*n* = 5), and 1.9% (*n* = 1) chose not to disclose their gender. Nurses dominate the sample (*n* = 46, 86.7%). Respondents from acute settings comprised 30.1% (*n* = 16), non-acute 50.9% (*n* = 27), and 18.7% (*n* = 10) work in both settings. Geographically, the North West (*n* = 17, 32.1%), Yorkshire and the Humber (*n* = 7, 13.2%), and the South West (*n* = 5, 9.4%) are well-represented although all regions of the UK were represented. Two responses were removed from the analysis due to either not being UK-based or not working as a specialist in infection control, and not having been involved in the OM process.

### Descriptive results

The survey contained both Likert and non-Likert scale questions related to the five broad outbreak management domains. Full responses to all Likert scale questions can be seen in Supplementary Information 2.

### Identification of outbreaks

#### Non-Likert scale questions

The majority of respondents (*n* = 27, 50.9%) indicated that they had no dedicated surveillance support within their teams.

#### Likert scale questions

There were four statements in the attitude scale for identification of outbreaks. The scale showed good internal consistency with a Cronbach’s Alpha coefficient of 0.84. Majorities agreed that they have robust processes to identify outbreaks (*n* = 44, 83.0%). 12 respondents (22.6%) indicated that they thought it likely that their organisation would fail to detect an outbreak, this correlated with confidence in timely detection of outbreaks for which 13 respondents (24.5%) felt their organisations do not identify outbreaks as early as possible. This may be explained by variance in perceptions of the robustness of surveillance support for which 16 respondents (30.2%) felt it was ineffective in their organisations.

### Outbreak investigation

#### Non-Likert scale questions

Most respondents (*n* = 35, 66.0%) indicated that they were aware of the number of IPC audits conducted during the last outbreak they managed. From this, (*n* = 33, 62.3%) reported a subsequent creation of an action plan and 47.2% (*n* = 25) reported that these action plans were followed up.

#### Likert scale questions

There were four statements in the attitude scale for outbreak investigation. The scale showed fair internal consistency with a Cronbach’s Alpha coefficient of 0.67. The majority of respondents (*n* = 41, 77.4%) agreed that the causes of outbreaks are always investigated. There were mixed responses to a statement regarding the causes of outbreaks with less than half (*n* = 25, 47.2%) indicating that they routinely determine the causes of outbreaks. Almost all respondents (*n* = 51, 96.2%) indicated that they were familiar with the processes used to investigate outbreaks. Most respondents (*n* = 35, 66.0%) agreed that the cause of outbreaks was considered important within outbreaks that they had been involved with.

### Case definition

There were no non-Likert scale questions for this domain.

#### Likert scale questions

There were three statements in the attitude scale for case definition. This scale showed a notably poor Cronbach’s Alpha coefficient of 0.29. Most respondents (*n* = 40, 75.5%) indicated that there is always a clear case definition for outbreaks they have been involved with. The majority (*n* = 42, 79.2%) indicated they felt confident formulating case definitions and agreed that case definitions are an essential part of outbreak management (*n* = 36, 67.9%).

### Control measures

#### Non-Likert scale questions

Most respondents (*n* = 52, 98.1%) indicated that they could state which IPC interventions were implemented during the last outbreak they managed. However, only 52.8% (*n* = 28) indicated that they could say how the efficacy of these interventions had been evaluated.

#### Likert scale questions

There were four statements in the attitude scale for control measures. The scale showed good internal consistency with a Cronbach’s Alpha coefficient of 0.86. The majority agreed that approaches to outbreak management planning had been systematic and well planned (*n* = 36, 67.9%). Just over half of respondents (*n* = 29, 54.7%) indicated that IPC interventions implemented as part of outbreak control efforts were well evaluated for effectiveness. However, most respondents (*n* = 39, 73.6%) indicated that they felt in control when managing outbreaks and 64.2% (*n* = 34) felt that staff in areas affected by outbreaks felt like the outbreaks are well controlled.

### Communication

#### Non-Likert scale questions

The majority (*n* = 42, 79.2%) indicated that a clearly documented outbreak management plan detailing control measures was produced during the most recent outbreak they were involved with. The majority of respondents indicated that an action log is kept tracking actions, staff assigned to complete the action and the action status (*n* = 33, 62.3%). 58.5 % (*n* = 31) of respondents indicated that a summary document was created containing all details of the outbreak management effort at the end of the outbreak.

#### Likert scale questions

There were three statements in the attitude scale for communication. The scale showed good internal consistency with a Cronbach’s Alpha coefficient of 0.82. Most respondents (*n* = 39, 73.6%) felt that information about outbreak management interventions are communicated effectively to relevant stakeholders. Most (*n* = 42, 79.2%) indicated that they felt knowledgeable about control efforts during ongoing outbreaks. Fewer (*n* = 39, 73.6%) felt that members of the outbreak control team effectively communicate between one another to manage outbreaks.

The last question of the survey offered respondents the opportunity to provide any additional information about their experiences of outbreak management. In total, 47.2% of respondents (*n* = 25) provided a free-text response. These responses were insufficient for robust qualitative analysis; however, they indicated issues related to currently employed approaches to outbreak management in the UK.

One respondent indicated that there is a lack of clarity around processes to support the outbreak management response:

‘There is no set process established currently that enables effective outbreak management, the processes and guidelines required are unclear and appear to be reactive rather than proactive. Outbreak management presently seems driven more by organisational pressures than patient safety.’

Some respondents suggested that there is apathy towards investment in developing the specialty of IPC which they felt hinders improvement of outbreak management processes.

‘The IPC team is vastly understaffed to enable effective education and support and daily interventions at the site of the infection. There has been no investment in surveillance software for infections despite repeated requests…without the adequate levels of IPC staff to implement training to be a preventative service we then become passive observers of outbreaks.’

### Freedom of information requests

In total, completed FOI responses were received for nine out of the 10 NHS trust contacted. One trust responded but did not provide the data, citing exemption under section 12 of the Freedom of Information Act and stating that it would be prohibitively expensive to retrieve the requested data, and not possible within 18 h. Details of the responses can be seen in Table 1.

**Table 1.** FOI response data.

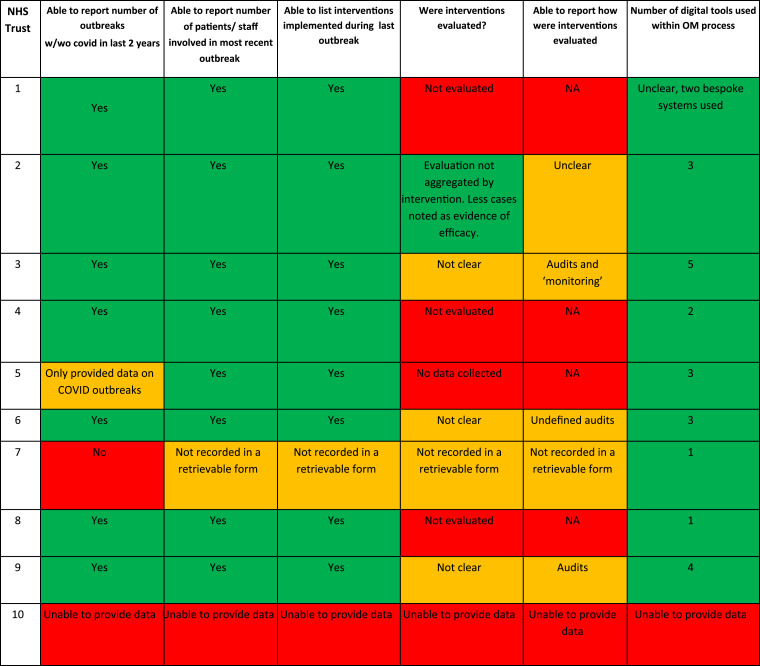

Red indicates that data are unavailable, amber than it is unclear if data are available and red indicates data are unavailable.

**Figure 1.**
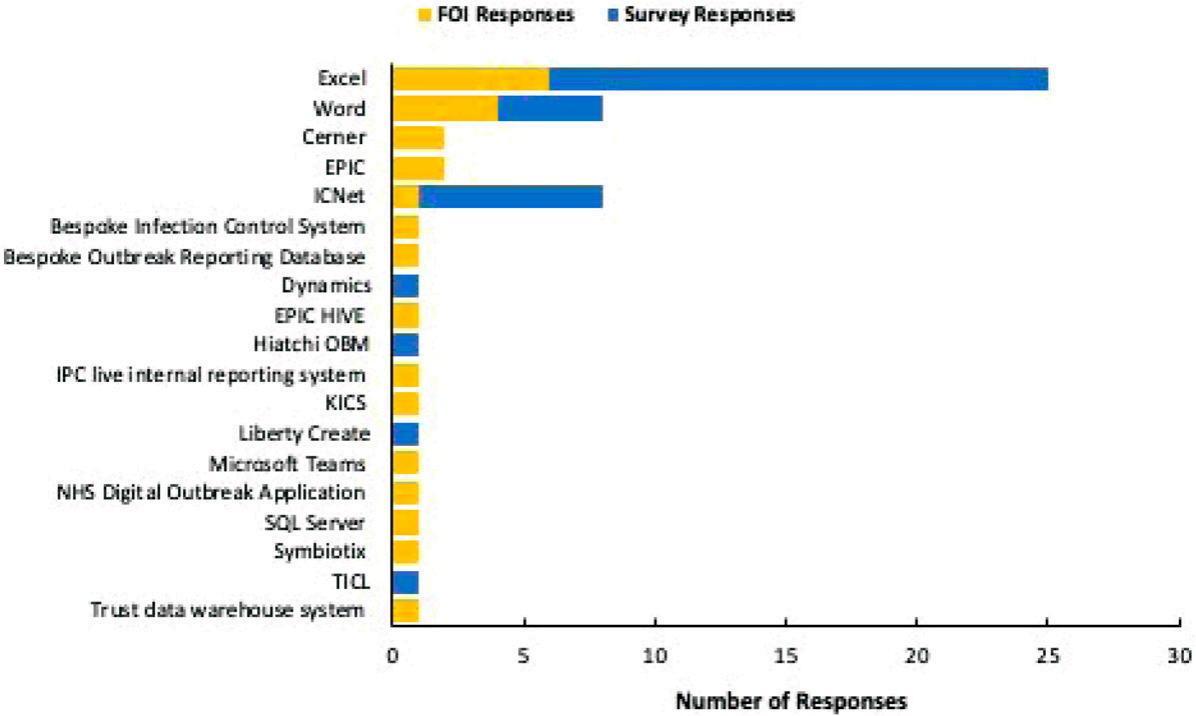
The number of NHS trusts/survey respondents identifying which digital tools are used within their OM processes.

Data were collected from both the FOI requests and the survey to identify any digital tools currently in use as part of the outbreak management process. Survey respondents were also asked to identify any frameworks or tools they used to manage outbreaks. Figure 1 illustrates the digital tools reported by both survey respondents and within FOI responses.

A total of nine (18.5%) survey respondents indicated that they currently use no tools or frameworks to assist with the production of outbreak management plans and to document decisions, with five survey respondents (9.4%) indicating they used paper-based tools.

## Discussion

This is the first study to examine outbreak management processes within UK health services. To the authors knowledge, this is also the first cross-sectional survey undertaken of the UK IPC workforce related to OM. There are currently no nationally recognised measures for assessing outbreak management efficacy. The field of outbreak management, from an operational perspective, is still relatively understudied, and little is known about the attitudes of and understanding held by practitioners in this domain. This study utilised data from both a survey and FOI requests. There were variations noted between responses to these differing data collection approaches.

Within survey responses, respondents held mixed perceptions of current outbreak management (OM) approaches. They generally viewed performance in the five key OM stages positively, for example, respondents typically reported that felt they have robust processes to identify outbreaks (*n* = 44, 83.0%), most (*n* = 41, 77.4%) agreed that the causes of outbreaks are always investigated, that approaches to outbreak management planning had been systematic and well planned (*n* = 36, 67.9%) and most respondents (*n* = 39, 73.6%) indicated that they felt in control when managing outbreaks. A specific attitude scale regarding case definitions showed poor internal reliability. Reversed directionality in a scale statement (see 26.j in Supplementary Information 2) may have led to misinterpretation. Other findings highlighted uncertainty in interventions' follow-up and audit effectiveness. Within free-text responses, respondents cited factors like unclear OM processes, surveillance limitations, and apathy towards IPC as a specialty.

Within FOI requests, a notable theme was confusion surrounding the interpretation of ‘intervention’ and ‘evaluation’. Responding organisations exhibited uncertainty in distinguishing between these concepts, making retrospective reviews challenging in OM. The use of epidemiological data to determine OM efficacy was limited, with only one trust reporting such practices. Although it is feasible, that OM teams may review epidemiological data, the FOI responses contradict the generally positive perceptions of data availability and outbreak control as reported in the survey. Although evaluation of outbreak interventions may be considered expensive and impractical (Pegorie et al., 2014), it may be facilitated if efforts are made to proactively collect additional quantitative or qualitative data to help determine if interventions were effective.

Regarding data to support OM, most respondents claimed awareness of interventions, diverging from FOI results which showed poorly documented and even inaccessible data relating to interventions. The FOI data revealed a diversity of interpretations of the term ‘intervention’, in some cases these were ambiguously defined, for example, ‘enhanced hand hygiene’ ‘monitoring by IPC experts’ and ‘situation reporting’ or even concept such as ‘duty of candour’. Interventions varied, with emphasis on cleaning, communication, and screening/isolation. IPC staff presence was reported as valuable. Few trusts explicitly evaluated intervention efficacy, often detailing audits, or use of published guidance as a measure of effectiveness. Thereby assuming the audited intervention itself is effective or that the guidance is appropriate in all situations within which it was applied. Full details of the reported OM interventions can be seen in Supplementary Information 3. In relation to basic data on the number of outbreaks, it was noted that four trusts were unable to provide the number of outbreaks they had experienced over the 2 years prior, indicating that documentation of outbreak occurrence and related intelligence is poor.

In relation to digitalisation of the OM process, the data from both the survey and FOI requests indicated that currently Microsoft Excel and Word are the most used digital tools. The use of which pose challenges as the unintended use of these applications introduces the potential for compromised data integrity, inaccuracy, and limits the utility of the recorded information. These non-standardised tools may lead to data fragmentation and hinder effective collaboration between healthcare organisations (Peng et al., 2020). To improve outbreak response quality, efforts are needed to establish a standardised approach to OM and a minimum data set requirement to enable retrospective review of outbreaks.

Healthcare authorities should consider adopting standardised digital platforms designed for real-time data sharing and interoperability, enabling swift and informed decision-making during outbreaks. By utilising standardised systems, health services could enhance preparedness and response capabilities, ultimately improving the effectiveness of outbreak management efforts. This issue was also identified in a recent study on electronic data collection in low and middle-income countries (Keating et al., 2021).

Finally, within FOI responses, it was noted that there were discrepancies in defining the term ‘outbreak’ across trusts, with one trust notably using ‘outbreak’, ‘cluster’, and ‘sporadic cases’ interchangeably. This lack of clarity may affect accurate outbreak reporting and hinder efforts to understand the true nature and scale of outbreaks.

Future research should focus on establishing a minimum data set required for effective outbreak management efforts, in addition to development of new digital tooling which can be integrated across organisations to facilitate learning from outbreaks. Further qualitative study is also needed to better understand the processes employed currently by teams responsible for OM to provide greater depth to our understanding of what influences perceptions of practice in this area among IPC practitioners.

### Limitations

The survey’s limited sample size might not fully capture nationwide practices, yet assessing significance is complex due to the absence of comprehensive data about the UK IPC workforce. Studies looking to understand the IPC workforce in the UK have typically used small samples, for example, an early study which utilised interviews with only four IPC leads in UK NHS trusts to understand IPC practices (Barrett et al., 2008). A more recent survey seeking to establish how IPC services best operate yielded only 70 responses (Burnett et al., 2023), in this case, the survey was distributed via the Infection Prevention Society, however, the inclusion criteria for this study were wider in that the respondents did not require experience in outbreak management.

FOI requests were only sent to the Shelford Group of NHS Trusts due to practicalities, but other smaller providers may have valuable contributions however this was beyond the scope of this study.

### Conclusion

Overall, the findings of this study indicate that there are currently challenges associated with approaches to OM within the UK healthcare system. Whilst the perceptions of IPC practitioners appear broadly to be positive towards the process, issues were identified. These include poor surveillance processes potentially leading to delayed outbreak detection and limiting the ability to evaluate intervention efficacy using epidemiological data. A lack of robust data collection and follow up of interventions and audits was reported. Limited digitalisation of the process was identified, with a dependence on non-standardised Microsoft Excel and Word-based tools, limiting the accessibility of robust data and therefore precluding the possibility for meaningful retrospective learning from outbreaks.

## Supplemental Material

Supplemental Material - Perceptions and digitalisation of outbreak management in UK health services: A cross-sectional survey Supplemental Material for Perceptions and digitalisation of outbreak management in UK health services: A cross-sectional survey by Matthew Wynn in Journal of Infection Prevention
