# Supplementary material for: Perceptions and digitalisation of outbreak management in UK health services: A cross-sectional survey
Source: J Infect Prev. 2024 Mar 19;25(4):134–41. doi: 10.1177/17571774241239221 (PMC11268242; doi:10.1177/17571774241239221)
Supplement: Supplemental Material - Perceptions and digitalisation of outbreak management in UK health services: A cross-sectional survey [file sj-pdf-1-bji-10.1177_17571774241239221.pdf]

Supplementary information 1. Survey respondent characteristics

Demographic	Number / proportion of sample
Age	
18-24	0 (0.0%)
25-34	5 (9.4%)
35-44	8 (15.1%)
45-54	20 (37.7%)
55-54	20 (37.7%)
65+	0 (0.0%)
Prefer not to say	0 (0.0%)
Years of experience in IPC	
1-5	10 (18.9%)
5-10	13 (24.5%)
10+	30 (56.6%)
Formal qualifications in IPC	
None	11 (20.8%)
RCN IPC course	4 (7.5%)
Diploma	7 (13.2%)
Bachelor's degree	4 (7.5%)
Post-graduate certificate	2 (3.8%)
Level 6 study	2 (3.8%)
Masters level study	8 (15.1%)
Masters degree	9 (17.0%)
Other non-university course	6 (11.3%)
Gender	
Male	5 (9.4%)
Female	47 (88.7%)
Other	0 (0.0%)
Prefer not to say	1 (1.9%)
Profession	
Nurse	46 (86.7%)
Clinical scientist	2 (3.8%)
Doctor	2 (3.8%)
Other	3 (5.7%)
Healthcare setting	
Acute	16 (30.1%)
Non-acute	27 (50.9%)
Both acute and non-acute	10 (18.7%)
Geographical Region	
North East	2 (3.8%)
North West	17 (32.1%)
Yorkshire and the Humber	7 (13.2%)
East Midlands	2 (3.8%)
West Midlands	3 (5.7%)
East of England	3 (5.7%)
London	3 (5.7%)
South East	3 (5.7%)
South West	5 (9.4%)
Wales	4 (7.5%)
Scotland	3 (5.7%)
Northern Ireland	1 (1.9%)

Supplementary information 2: Heat map of Likert scale responses

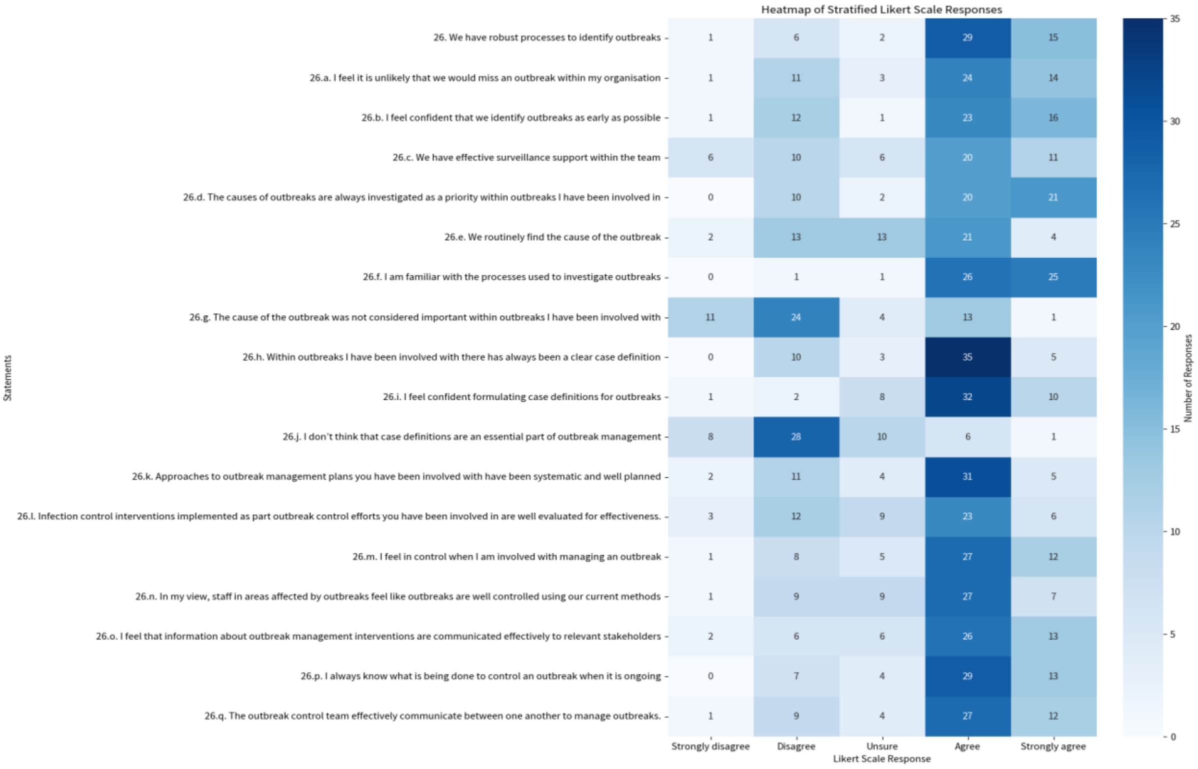

Supplementary information 3: Outbreak interventions reported within FOI responses

Outbreak interventions
Isolation of positive and symptomatic cases
PCR* testing of symptomatic cases
Cohorting of positive cases
Contact tracing
Visitor cessation
Duty of candour
Daily monitoring by IPC experts
Outbreak meetings
Electronic audit of respiratory assessments
Overview monitoring of outbreaks and submission to national databases
Audit of PPE* compliance
Decontamination of the environment
Restriction of staff movement / staff off work
CDT* audit for each case
Antibiotic audit
Enhanced cleaning using Chlorine releasing agent
Terminal cleans (including curtain change) of bed areas vacated
Screening of symptomatic staff
Outbreak escalated / communicated to patient flow and on-call teams
SitRep of outbreak through chief nurse DIPC* / COVID-19 Management group to NHSE/I*
Ward based hand hygiene and PPE audits daily
Environmental cleaning spot checks weekly
All staff are wearing FFP3* masks
Enhanced cleaning is in place
Ward closed
No sharing of catering between wards

Virucidal alcohol hand gel
Ward deep clean
Staff education
Support from IPC team
Root cause analysis
Source identified
Daily review of all symptomatic patients
Decontamination of reusable equipment and ward environment
COVID-19 safety checklist
Increased IPC and senior team presence
Individual post infection reviews and PII*
Reviewed IV* and device management practices
Ward communications to relatives
Promotion of ventilation
Correct placement of patient
Enhanced cleaning
Enhanced hand hygiene
Increasing full infection and control precautions across affected area

Notes:

1-Two NHS trusts were unable to provide data on the interventions implemented during the last outbreak they experienced.

2-The compilation of this list is solely derived from the data provided through FOI requests and does not indicate the authors' validation of the listed interventions.

*Acronyms have been reported as they were within FOI responses

Supplementary information 4. CROSS checklist

Checklist for Reporting Of Survey Studies (CROSS)

Section/topic	Item	Item description	Reported on page #
**Title and abstract**			**1**
Title and abstract	1a	State the word "survey" along with a commonly used term in title or abstract to introduce the study's design.	1
	1b	Provide an informative summary in the abstract, covering background, objectives, methods, findings/results, interpretation/discussion, and conclusions.	1
**Introduction**			
Background	2	Provide a background about the rationale of study, what has been previously done, and why this survey is needed.	2
Purpose/aim	3	Identify specific purposes, aims, goals, or objectives of the study.	3
**Methods**			
Study design	4	Specify the study design in the methods section with a commonly used term (e.g., cross-sectional or longitudinal).	3
Data collection methods	5a	Describe the questionnaire (e.g., number of sections, number of questions, number and names of instruments used).	3
	5b	Describe all questionnaire instruments that were used in the survey to measure particular concepts. Report target population, reported validity and reliability information, scoring/classification procedure, and reference links (if any).	3-5

	5c	Provide information on pretesting of the questionnaire, if performed (in the article or in an online supplement). Report the method of pretesting, number of times questionnaire was pre-tested, number and demographics of participants used for pretesting, and the level of similarity of demographics between pre-testing participants and sample population.	4
	5d	Questionnaire if possible, should be fully provided (in the article, or as appendices or as an online supplement).	Included as supplement
Sample characteristics	6a	Describe the study population (i.e., background, locations, eligibility criteria for participant inclusion in survey, exclusion criteria).	4 / supplement
	6b	Describe the sampling techniques used (e.g., single stage or multistage sampling, simple random sampling, stratified sampling, cluster sampling, convenience sampling). Specify the locations of sample participants whenever clustered sampling was applied.	4-5
	6c	Provide information on sample size, along with details of sample size calculation.	4-5 / supplement
	6d	Describe how representative the sample is of the study population (or target population if possible), particularly for population-based surveys.	4 / 13
Survey administration	7a	Provide information on modes of questionnaire administration, including the type and number of contacts, the location where the survey was conducted (e.g., outpatient room or by use of online tools, such as SurveyMonkey).	5
	7b	Provide information of survey's time frame, such as periods of recruitment, exposure, and follow-up days.	5
	7c	Provide information on the entry process:<br>–>For non-web-based surveys, provide approaches to minimize human error in data entry.<br>–>For web-based surveys, provide approaches to prevent "multiple participation" of participants.	5<br>N/A survey could only be completed once
Study preparation	8	Describe any preparation process before conducting the survey (e.g., interviewers' training process, advertising the survey).	NA
Ethical considerations	9a	Provide information on ethical approval for the survey if obtained, including informed consent, institutional review board [IRB] approval, Helsinki declaration, and good clinical practice [GCP] declaration (as appropriate).	5
	9b	Provide information about survey anonymity and confidentiality and describe what mechanisms were used to protect unauthorized access.	5
Statistical analysis	10a	Describe statistical methods and analytical approach. Report the statistical software that was used for data analysis.	5
	10b	Report any modification of variables used in the analysis, along with reference (if available).	NA
	10c	Report details about how missing data was handled. Include rate of missing items, missing data mechanism (i.e., missing completely at random [MCAR], missing at random [MAR] or missing not at random [MNAR]) and methods used to deal with missing data (e.g., multiple imputation).	NA
	10d	State how non-response error was addressed.	NA
	10e	For longitudinal surveys, state how loss to follow-up was addressed.	NA

	10f	Indicate whether any methods such as weighting of items or propensity scores have been used to adjust for non-representativeness of the sample.	NA
	10g	Describe any sensitivity analysis conducted.	NA
**Results**			
Respondent characteristics	11a	Report numbers of individuals at each stage of the study. Consider using a flow diagram, if possible.	6
	11b	Provide reasons for non-participation at each stage, if possible.	NA
	11c	Report response rate, present the definition of response rate or the formula used to calculate response rate.	NA
	11d	Provide information to define how unique visitors are determined. Report number of unique visitors along with relevant proportions (e.g., view proportion, participation proportion, completion proportion).	NA
Descriptive results	12	Provide characteristics of study participants, as well as information on potential confounders and assessed outcomes.	In supplementary information
Main findings	13a	Give unadjusted estimates and, if applicable, confounder-adjusted estimates along with 95% confidence intervals and p-values.	NA
	13b	For multivariable analysis, provide information on the model building process, model fit statistics, and model assumptions (as appropriate).	NA
	13c	Provide details about any sensitivity analysis performed. If there are considerable amount of missing data, report sensitivity analyses comparing the results of complete cases with that of the imputed dataset (if possible).	NA
**Discussion**			
Limitations	14	Discuss the limitations of the study, considering sources of potential biases and imprecisions, such as non-representativeness of sample, study design, important uncontrolled confounders.	13
Interpretations	15	Give a cautious overall interpretation of results, based on potential biases and imprecisions and suggest areas for future research.	13-14
Generalizability	16	Discuss the external validity of the results.	14
**Other sections**			
Role of funding source	17	State whether any funding organization has had any roles in the survey's design, implementation, and analysis.	16
Conflict of interest	18	Declare any potential conflict of interest.	16
Acknowledgements	19	Provide names of organizations/persons that are acknowledged along with their contribution to the research.	16

Supplementary information 5. Survey tool administered digitally via JISC survey tool

Consent form

1. I confirm that I have read the information sheet [V2 11/01/2023] for this study on the previous page.

2. I have had the opportunity to consider the information, ask questions and have had any questions answered satisfactorily.
3. I understand that my participation is voluntary and that I am free to withdraw at any time before submitting the results without giving any reason.
4. I understand that the information collected about me will be used to support other research in the future and may be shared anonymously with other researchers.
5. I understand if I give my contact details they will be stored securely by the research team for 5 years for the purpose of sharing progress and inviting participation in follow up activity related to this project.
6. I agree to take part in the above study.

Demographic details

7. What is your age?
8. How many years of experience in infection control do you have?
9. Please specify any formal qualifications you have in infection control. If you have none, please write 'none'.
10. What is your gender?
11. Please specify your profession.

11.a. If you selected Other, please specify:

Please specify your role.

12. Have you ever been involved in managing an outbreak?
13. Do you currently use a tool or framework to assist with the production of outbreak management plans/to document decisions? (e.g. software. Excel, paper-based tool). If the answer is no please write 'no' if yes, please specify.
14. Do you work in an acute or non-acute setting?
15. Which region do you work in?

Non-Likert scale questions

The following questions relate to the availability of outbreak management data within your IPC service.

Please assess honestly whether you could access the following data related to the most recent confirmed outbreak you were involved with:

16. Was a clearly documented outbreak management plan produced describing all outbreak control measures implemented during the outbreak?
17. Do you have dedicated surveillance support within your team?
18. If asked to, could you state which IPC interventions were implemented during this outbreak?
19. If asked to, could you state how these interventions were evaluated?
20. Can you state the number of IPC audits conducted during the outbreak. (e.g., hand hygiene, personal protective equipment, environmental)?
21. Were all these audits followed up with an action plan?
22. Is there evidence of these plans being followed up?

23. Was there an action log created to keep track of actions, staff assigned and action status?
24. Is there a summary document for example, a document containing all details of the outbreak management effort including epidemiological data and interventions implemented, produced at the end of the outbreak?

Likert Scale statements
(related to identification of outbreaks)*
26. We have robust processes to identify outbreaks.
26.a. I feel it is unlikely that we would miss an outbreak within my organisation.
26.b. I feel confident that we identify outbreaks as early as possible.
26.c. We have effective surveillance support within the team.
(related to investigation of outbreaks)*
26.d. The causes of outbreaks are always investigated as a priority within outbreaks I have been involved in.
26.e. We routinely find the cause of the outbreak.
26.f. I am familiar with the processes used to investigate outbreaks.
26.g. The cause of the outbreak was not considered important within outbreaks I have been involved with.
(related to development of case definition)*
26.h. Within outbreaks I have been involved with there has always been a clear case definition.
26.i. I feel confident formulating case definitions for outbreaks.
26.j. I don’t think that case definitions are an essential part of outbreak management.
(related to control of outbreaks)*
26.k. Approaches to outbreak management plans you have been involved with have been systematic and well planned.
26.l. Infection control interventions implemented as part outbreak control efforts you have been involved in are well evaluated for effectiveness.
26.m. I feel in control when I am involved with managing an outbreak.
26.n. In my view, staff in areas affected by outbreaks feel like outbreaks are well controlled using our current methods.
(related to communication surrounding outbreak management)*
26.o. I feel that information about outbreak management interventions are communicated effectively to relevant stakeholders.
26.p. I always know what is being done to control an outbreak when it is ongoing.
26.q. The outbreak control team effectively communicate between one another to manage outbreaks.

27. Please use this space to add any additional information about your experiences of outbreak management which you feel may be relevant. (free text)

***text in brackets was not visible to respondents but illustrates how attitude scales were constructed for each outbreak management phase.**

Supplementary information 6. FOI request

I am writing to make a request under the Freedom of Information Act 2000 for information about how your Trust has managed outbreaks of communicable disease.

Specifically, I would like to request the following information:

1. The total number of communicable disease outbreaks (by organism / resistance mechanism) that have occurred within the Trust over the past two years.
2. For the most recent, concluded outbreak, I would like to know the number of staff and patients who were involved.
3. For the most recent, concluded outbreak, please provide the details of the interventions that were put in place to manage the outbreak, including (but not limited to) any infection control measures, isolation procedures, and contact tracing activities.
4. For the most recent, concluded outbreak, please state how the interventions were assessed for effectiveness, including any data or analysis that was used to evaluate their success.
5. Please state any digital tools you use as part of your outbreak management processes. This might include, for example, Word, Excel, ICNet.

I have provided an example data collection table below. Please indicate explicitly, as stated in the table, if data cannot be obtained within the 18 hours because of how it is collected or if the data is not collected.

Data collection table

Number of communicable disease outbreaks (by organism / resistance mechanism that have occurred within the Trust over the last two years		For the most recent outbreak,<br><br>The number of staff and patients who were involved	For the most recent outbreak<br><br>Details of the interventions which were put in place	For the most recent outbreak<br><br>The effectiveness of each intervention	Digital tools used as part of outbreak management processes
Organism	Number of outbreaks	Number of staff:<br><br>Number of patients:	List of interventions:<br>1-<br>2-<br>3-	Evaluation method for intervention:<br>1-<br>2-<br>3-	Digital tool:<br>1-<br>2-<br>3-
This data is not collected in a way which can be accessed within 18 hours		This data is not collected in a way which can be accessed within 18 hours	This data is not collected in a way which can be accessed within 18 hours	This data is not collected in a way which can be accessed within 18 hours	We do not use digital tools as part of our outbreak control processes
This data is not collected		This data is not collected	This data is not collected	This data is not collected	N/A

Please provide the information in an electronic format if possible, and within the 20 working day deadline set out by the Freedom of Information Act. If you need any clarification or further information to assist you in providing this information, please let me know as soon as possible.

Thank you for your attention to this matter.

Yours faithfully,
